# Reducing effects of isoliquiritigenin, a naturally occurring GABA_B_ receptor agonist, on alcohol-motivated behaviors in alcohol-preferring rats

**DOI:** 10.1007/s00213-025-06892-x

**Published:** 2025-09-23

**Authors:** Paola Maccioni, Laura Regonini Somenzi, Mauro A. M. Carai, Federico Corelli, Gian Luigi Gessa, Giancarlo Colombo

**Affiliations:** 1 Neuroscience Institute, Section of Cagliari, National Research Council of Italy, S.S. 55, km. 4,500, I-09042 Monserrato, CA Italy; 2 Cagliari Pharmacological Research, Cagliari, CA Italy; 3 Department of Biotechnology, Chemistry, and Pharmacy, University of Siena, Siena, SI Italy

**Keywords:** Isoliquiritigenin, *Glycyrrhiza glabra* L., Licorice, GABA_B_ receptor, Baclofen, Alcohol self-administration, Reinstatement of alcohol seeking, Alcohol-preferring rats

## Abstract

**Rationale and objectives:**

*Glycyrrhiza glabra* L. (Fabaceae; licorice) is a widely used medicinal herb known to exert protective effects against multiple neurological diseases. The flavonoid, isoliquiritigenin (ISL), is a main constituent of roots of *Glycyrrhiza glabra*. ISL has been reported to behave as a GABA_B_ receptor agonist and exert multiple pharmacological effects. Given the role of the GABA_B_ receptor in the neurobiological and pharmacological bases of alcohol use disorder, the present study investigated the effect of ISL on a series of alcohol-related behaviors in selectively bred, female Sardinian alcohol-preferring rats.

**Methods and results:**

The collected results indicated that acute treatment with ISL (5-20 mg/kg, i.p.; 50-200 mg/kg, i.g.) decreased operant oral alcohol self-administration under both fixed and progressive ratio schedules of reinforcement and suppressed cue-induced reinstatement of alcohol seeking. ISL effect on alcohol self-administration was partially blocked by pretreatment with the GABA_B_ receptor antagonist, SCH50911, and potentiated by co-administration of the positive allosteric modulator of the GABA_B_ receptor, GS39783. Acute treatment with doses of ISL as high as 80 mg/kg (i.p.) did not alter spontaneous locomotor activity, suggestive of the specificity of ISL effects on alcohol-related behaviors.

**Conclusions:**

These results confirm the ability of ISL to behave *in vivo* as a GABA_B_ receptor agonist; they also indicate that ISL reproduced the suppressing effects of the prototypic GABA_B_ receptor agonist, baclofen, on multiple alcohol-related behaviors in rodents.

## Introduction

*Glycyrrhiza glabra* L. (Fabaceae; licorice) is one of the oldest and most widely used medicinal herbs in several traditional medicines. Ethnopharmacological information suggests that preparations based on *Glycyrrhiza glabra* exert protective effects against multiple neurological diseases, including long-term depression, cognitive deficits, anxiety, Alzheimer’s disease, epilepsy, and drug addiction (see Sharma et al. 2023).

**Fig. 1:**
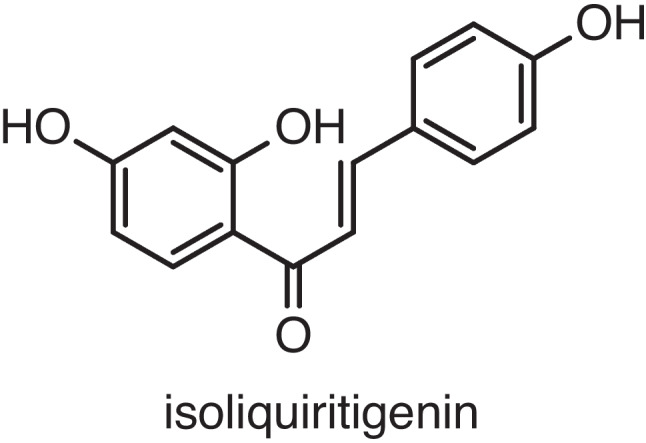
Chemical structure of isoliquiritigenin (ISL)

The flavonoid, isoliquiritigenin (ISL; Fig. 1), is one of the main active constituents of *Glycyrrhiza glabra* roots (see Wahab et al. 2021). ISL has been reported to exert a series of pharmacological effects, including antineoplastic activity, neuroprotection, memory enhancement, and anti-inflammation (see Peng et al. 2015; Ramalingam et al. 2018; Wang et al. 2021; Mustafa et al. 2025). A recent molecular docking study – i.e., a computational approach used nowadays to effectively predict the molecular targets of natural compounds (see Thomford et al. 2018) – indicated that ISL had interactions with the GABA_B_ receptor and suggested that ISL may behave as a GABA_B_ receptor ligand (Lin et al. 2021). More specifically, ISL established (i) hydrogen-bonding interactions with the amino acid residue Glu349, (ii) a van der Waal lipophilic interaction with the amino acid residue His170, and (iii) a lipophilic interaction with the amino acid residue Cys129 of the GABA_B_-receptor structure. Notably, His170 and Glu 349 are major residues of LB1 domain, the portion of the GABA_B_ receptor essential for agonist recognition and binding (Lin et al. 2021).

Consonant with the results of this docking study, several *in vivo* and *ex-vivo* studies demonstrated that specific pharmacological effects of ISL are indeed mediated by activation of the GABA_B_ receptor. First, pretreatment with the GABA_B_ receptor antagonist, SCH50911, prevented ISL-induced blockade of cocaine-stimulated dopamine release in the rat nucleus accumbens (Jang et al. 2008). Second, co-application of the GABA_B_ receptor antagonist, CGP35348, prevented ISL-induced inhibition of glutamate release in rat cerebrocortical synaptosomes (Lin et al. 2021). These data find an interesting extension in a study testing a methanolic extract of *Glycyrrhiza glabra* roots: extract-induced prevention of locomotor sensitization produced by methamphetamine in rats was abolished by pretreatment with SCH50911 (Zhao et al. 2014a), thus suggesting that the extract ingredient(s) responsible for this effect acted *via* a GABA_B_ receptor-mediated mechanism.

Based on these premises, suggestive of an agonistic activity of ISL at the GABA_B_ receptor, it is of interest to assess whether and to which extent ISL reproduces the composite pharmacological profile of synthetic GABA_B_ receptor agonists. Bearing this in mind, the present study investigated the ability of ISL to replicate the effects of the prototypic GABA_B_ receptor agonist, baclofen, on different alcohol-motivated behaviors in rats. Treatment with baclofen has indeed been repeatedly reported to suppress excessive alcohol drinking, including binge- and relapse-like drinking, operant oral alcohol self-administration, reinstatement of alcohol-seeking behavior, alcohol-stimulated locomotor activity, and alcohol-induced conditioned place preference in laboratory rodents and non-human primates (see Colombo and Gessa 2018; Holtyn and Weerts 2022). These experimental results have subsequently been extended to patients affected by alcohol use disorder: treatment with baclofen effectively reduced alcohol drinking and craving for alcohol, thus promoting abstinence and preventing the risk of relapse (see Agabio et al. 2023, 2024).

More specifically, the present study investigated the effect of acute treatment with ISL on operant oral alcohol self-administration [measure of the reinforcing and motivational properties of alcohol (see Markou et al. 1993)] and reinstatement of alcohol seeking [experimental model of human loss of control over alcohol and relapse into heavy drinking (see Martin-Fardon and Weiss 2013)] in selectively bred, Sardinian alcohol-preferring (sP) rats (see Colombo et al. 2006). Selectivity and specificity of ISL effect on alcohol self-administration were assessed investigating the impact of acute treatment with ISL on operant oral self-administration of an alternative, non-drug reinforcer (sucrose solution) and spontaneous locomotor activity, respectively. Finally, the contribution of the GABA_B_ receptor to ISL effect on alcohol self-administration was assessed by investigating whether pretreatment with SCH50911 blocked, and combination with the positive allosteric modulator of the GABA_B_ receptor GS39783 potentiated, ISL effect on alcohol self-administration.

## Materials and methods

The experimental procedures employed in the present study fully complied with European Directive no. 2010/63/EU and subsequent Italian Legislative Decree no. 26, March 4, 2014, on the “Protection of animals used for scientific purposes”.

### Animals

Female sP rats (bred in house) were used. Rats were 60-days-old at the start of each experiment, from the 121^st^ and 122^nd^ generations, and alcohol-naive at the start of each experiment. Rats were housed 3 per cage in standard plastic cages with wood chip bedding. The animal facility was under an inverted 12:12-hour light-dark cycle (lights on at 05:00 p.m.), at a constant temperature of 22 ± 2 °C and relative humidity of approximately 60%. Standard rat chow and tap water were always available in the homecage, except as noted below. Rats were extensively habituated to handling, intraperitoneal (i.p.) injections, and intragastric (i.g.) infusions (the latter limited to rats allocated to Experiments 1 C and 1D).

The main reason for choosing female rats over male rats lies in the fact that body weight is more stable and much lower in adult female than male sP rats, resulting in the several practical advantages described elsewhere (Lorrai et al. 2019). Taking into account the aims of the present study, we note that sensitivity of alcohol self-administration to pharmacological manipulation of the GABA_B_ receptor is highly comparable in female and male sP rats (e.g.: Lorrai et al. 2019).

Each single experiment used an independent set of rats. Sample size for each single experiment was calculated using G*Power 3.1.9.7 software (Faul et al. 2007).

### Drugs

ISL (Biosynth Ltd., Bratislava, Slovakia) was suspended in saline with Tween 80 (5% w/v) and polyethylene glycol 400 (3% w/v) and administered either i.p. at an injection volume of 2 ml/kg or i.g. at an infusion volume of 6 ml/kg. SCH50911 (Tocris Bioscience, Bristol, UK) was dissolved in saline and administered i.p. at an injection volume of 2 ml/kg. GS39783 (synthetized in house by FC) was suspended in distilled water with a few drops of Tween 80 and administered i.g. at an infusion volume of 2 ml/kg. Drug doses and pretreatment times are detailed below.

### Alcohol self-administration and cue-induced reinstatement of alcohol seeking

#### Apparatus

Self-administration, extinction-responding, and reinstatement sessions were conducted in modular chambers (Med Associates, St. Albans, VT, USA) described in detail elsewhere (e.g.: Maccioni et al. 2015). Briefly, each chamber was equipped with 2 retractable response levers (connected to 2 syringe pumps located outside the chamber), one dual-cup liquid receptacle, 2 stimulus lights (mounted above each lever), and one tone generator.

In self-administration sessions, achievement of the response requirement (RR) had the following consequences: activation of alcohol (or sucrose) or water pumps, delivery of 0.1 ml fluid, illumination of the stimulus light for the time period of fluid delivery, and activation of the tone generator.

#### Experimental procedure

##### Training and maintenance phases of alcohol or sucrose self-administration

In alcohol self-administration experiments, rats were initially exposed to the homecage 2-bottle “alcohol (10% v/v) *vs* water” choice regimen with unlimited access for 24 hours/day over 10 consecutive days, according to the procedure described in detail elsewhere (e.g.: Maccioni et al. 2015). Subsequently, rats were introduced into the operant chambers and trained to lever-respond for alcohol. Self-administration sessions lasted 30 min (with the sole exception of the very first session, that lasted 120 min) and were conducted 5 days per week. Rats were water-deprived exclusively during the 12 hours prior to the first session in the operant chamber. Rats were initially exposed to a Fixed Ratio (FR) 1 (FR1) schedule of reinforcement for 10% alcohol (v/v) for 4 sessions. FR was then progressively increased to FR5 over 4 sessions. In sessions 9 and 10, the alcohol solution was presented at a final concentration of 15% (v/v). Rats were then exposed to 4 sessions during which the water lever alone or alcohol lever alone was available every other day; water and alcohol were available on FR1 and FR5, respectively. From then onwards, both levers were concomitantly available (maintenance phase) for a total of 20 sessions conducted with FR5 and FR1 on the alcohol and water lever, respectively. On completion of the maintenance phase, rats displaying the most stable responding behavior were selected for use in the “alcohol” portion of Experiments 1 A, 1D, and 2, as well as in Experiments 1B, 1 C, and 3.

In sucrose self-administration experiments, rats were trained to lever-respond for a sucrose solution. Self-administration sessions lasted 30 min (with the sole exception of the very first session, that lasted 120 min) and were conducted 5 days per week. Rats were water-deprived exclusively during the 12 hours prior to the first session in the operant chamber. Rats were initially exposed to an FR1 schedule of reinforcement for 2% (w/v) sucrose solution (in tap water) for 4 sessions. FR was then progressively increased to FR5 over 4 sessions. Sucrose concentration was reduced to 1% (w/v) over 6 sessions. This sucrose concentration was selected as to equate lever-responding for alcohol recorded in the rat groups used in the corresponding “alcohol” experiments. Rats were then exposed to 4 sessions during which the water lever alone or the sucrose lever alone was available every other day; water and sucrose were available on FR1 and FR5, respectively. From then onward, both levers were concomitantly available (maintenance phase) for a total of 20 sessions conducted with FR5 and FR1 on the sucrose and water lever, respectively. On completion of the maintenance phase, the rats displaying the most stable responding behavior were selected for use in the “sucrose” portion of Experiments 1 A, 1D, and 2.

##### Testing under the FR schedule

Experiment 1 A evaluated the effect of acute and systemic treatment with different doses of ISL on alcohol or sucrose self-administration under the FR schedule of reinforcement. ISL was administered i.p. at doses of 0, 5, 10, and 20 mg/kg, 30 min before start of the self-administration session. ISL dose range was chosen as to be identical to that previously tested on cocaine-induced increase in accumbal extracellular levels of dopamine (Jang et al. 2008), i.e. one of the few studies investigating an *in vivo* effect of ISL in rats. This ISL dose range was then also used in Experiments 2 and 3. The test session occurred the day after completion of the maintenance phase, lasted 30 min, and was identical to those of the maintenance phase [FR5 and FR1 on the alcohol (or sucrose) and water lever, respectively]. The “alcohol” experiment employed a total of *n*=56 rats, divided into 4 groups of *n*=14 matched for number of responses on the alcohol lever over the last 3 sessions of the maintenance phase; the “sucrose” experiment employed a total of *n*=64 rats, divided into 4 groups of *n*=16 matched for number of responses on the sucrose lever over the last 3 sessions of the maintenance phase.

Experiment 1B evaluated the effect of acute pretreatment with SCH50911 on the effect of acutely administered ISL on alcohol self-administration under the FR schedule of reinforcement. Namely, the following 4 treatment combinations were tested: 0 mg/kg SCH50911 + 0 mg/kg ISL; 2 mg/kg SCH50911 + 0 mg/kg ISL; 0 mg/kg SCH50911 + 10 mg/kg ISL; 2 mg/kg SCH50911 + 10 mg/kg ISL. SCH50911 was administered i.p. 5 min before treatment with ISL; ISL was administered i.p. 30 min before start of the self-administration session. SCH50911 dose (2 mg/kg) was chosen as totally ineffective, when given alone, on alcohol self-administration in sP rats (e.g.: Maccioni et al. 2016, 2017). ISL dose (10 mg/kg) was chosen on the basis of the results of Experiment 1 A as it produced a remarkable reduction in number of lever-responses for alcohol and amount of self-administered alcohol. The test session occurred the day after completion of the maintenance phase, lasted 30 min, and was identical to those of the maintenance phase (FR5 and FR1 on the alcohol and water lever, respectively). This experiment employed a total of *n*=60 rats, divided into 4 groups of *n*=15 matched for number of responses on the alcohol lever over the last 3 sessions of the maintenance phase.

Experiment 1 C evaluated the effect of the acute combination of GS39783 and ISL on alcohol self-administration under the FR schedule of reinforcement. Namely, the following 4 treatment combinations were tested: 0 mg/kg GS39783 + 0 mg/kg ISL; 0 mg/kg GS39783 + 5 mg/kg ISL; 5 mg/kg GS39783 + 0 mg/kg ISL; 5 mg/kg GS39783 + 5 mg/kg ISL. GS39783 was administered i.g. 5 min before treatment with ISL; ISL was administered i.p. 30 min before start of the self-administration session. GS39783 dose (5 mg/kg) was chosen as totally ineffective, when given alone, on alcohol self-administration but highly effective in potentiating the suppressing effect of baclofen on alcohol self-administration in sP rats (Maccioni et al. 2015). ISL dose (5 mg/kg) was chosen on the basis of the results of Experiment 1 A as totally ineffective, when given alone, on alcohol self-administration in sP rats. The test session occurred the day after completion of the maintenance phase, lasted 30 min, and was identical to those of the maintenance phase (FR5 and FR1 on the alcohol and water lever, respectively). This experiment employed a total of *n*=56 rats, divided into 4 groups of *n*=14 matched for number of responses on the alcohol lever over the last 3 sessions of the maintenance phase.

Experiment 1D evaluated the effect of acute and *per os* treatment with different doses of ISL on alcohol or sucrose self-administration under the FR schedule of reinforcement. ISL was administered i.g. at doses of 0, 50, 100, and 200 mg/kg, 30 min before the start of the self-administration session. ISL dose range was chosen on the basis of the results of a series of preliminary experiments aimed at identifying in sP rats the minimum effective dose of ISL after i.g. administration (this laboratory, unpublished results). The test session occurred the day after completion of the maintenance phase, lasted 30 min, and was identical to those of the maintenance phase [FR5 and FR1 on the alcohol (or sucrose) and water lever, respectively]. The “alcohol” experiment employed a total of *n*=40 rats, divided into 4 groups of *n*=10 matched for number of responses on the alcohol lever over the last 3 sessions of the maintenance phase; the “sucrose” experiment employed a total of *n*=28 rats, divided into 4 groups of *n*=7 matched for number of responses on the sucrose lever over the last 3 sessions of the maintenance phase.

Measured variables in Experiments 1A-D were (i) number of responses on each lever and (ii) amount of self-administered alcohol (expressed in g/kg pure alcohol) or sucrose solution (expressed in ml/kg), estimated from the number of earned reinforcers and assuming that each reinforcer was entirely consumed. In Experiments 1 A and 1D, latency (expressed in s) to the first alcohol (or sucrose) reinforcer was also measured; rats that not reach the RR for the first reinforcer were assigned the value 1800 s (i.e., the entire length of the test session). When normally distributed, data were analyzed by 1-way ANOVA with repeated measures, followed by Tukey’s test for *post hoc* comparisons; when not normally distributed, data were analyzed by Kruskal-Wallis.

##### Testing under the PR schedule

Experiment 2 evaluated the effect of acute and systemic treatment with different doses of ISL on alcohol or sucrose self-administration under a Progressive Ratio (PR) schedule of reinforcement. ISL was administered i.p. at doses of 0, 5, 10, and 20 mg/kg, 30 min before start of the self-administration session. ISL dose range was chosen as to be identical to that tested in Experiments 1 A and 3. The test session occurred the day after completion of the maintenance phase and lasted 60 min. In the test session, RR on the alcohol (or sucrose) lever was increased progressively over the session according to a procedure slightly adapted from that described by Richardson and Roberts (1996); Namely, RR was increased as follows: 5, 9, 12, 15, 20, 25, 32, 40, 50, 62, 77, 95, 118, 145, 178, 219, etc. The water lever was inactive. The “alcohol” experiment employed a total of *n*=48 rats, divided into 4 groups of *n*=12 matched for number of responses on the alcohol lever over the last 3 sessions of the maintenance phase; the “sucrose” experiment employed a total of *n*=40 rats, divided into 4 groups of *n*=10 matched for number of responses on the sucrose lever over the last 3 sessions of the maintenance phase.

Measured variables in Experiment 2 were (i) number of responses on each lever, (ii) breakpoint for alcohol (or sucrose), defined as the lowest RR not achieved by the rat, and (iii) latency (expressed in s) to the first alcohol (or sucrose) reinforcer. When normally distributed, data were analyzed by 1-way ANOVA with repeated measures, followed by Tukey’s test for *post hoc* comparisons; when not normally distributed, data were analyzed by Kruskal-Wallis, followed by Dunn’s test for *post hoc* comparisons.

##### Testing under the reinstatement of alcohol-seeking protocol

Experiment 3 evaluated the effect of acute and systemic treatment with different doses of ISL on cue-induced reinstatement of alcohol seeking. To this end, immediately after completion of the maintenance phase, rats underwent an extinction-responding phase made up of consecutive (no weekend interruption) daily sessions (lasting 60 min) characterized by unavailability of alcohol and water; specifically, syringe pumps, stimulus lights, and tone generator were off, and lever-responding was unreinforced. An extinction criterion was set at ≤12 responses on the alcohol lever per session for 2 consecutive sessions (Maccioni et al. 2019).

This experiment employed a total of *n*=48 rats, divided into 4 groups of *n*=12 matched for number of responses on the alcohol lever over the first 3 sessions of the extinction-responding phase. The day after achievement of the extinction criterion, each rat was exposed to a single 60-min reinstatement (test) session, during which a stimulus complex – previously associated to availability of alcohol – was presented for 10 times within 20 s. This stimulus complex was composed of tone, turning on of stimulus lights, and availability, every other time, of 0.1 ml alcohol (15% v/v) in the liquid receptacle (for a total number of 5 presentations). Immediately after the last presentation of the stimulus complex, both levers were inserted inside the chamber and lever-responding (still unreinforced) was recorded. ISL was administered i.p. at doses of 0, 5, 10, and 20 mg/kg, 30 min before start of the reinstatement session. ISL dose range was chosen to be identical to that tested in Experiments 1 A and 2.

The measured variable in Experiment 3 was number of responses on alcohol lever during the reinstatement session. Data were statistically evaluated by 2-way [phase (extinction/reinstatement); treatment (ISL dose)] ANOVA with repeated measures on the factor “phase”, followed by Sidak’s test for *post hoc* comparisons. An additional analysis evaluated the number of sessions of the extinction responding phase needed to achieve the extinction criterion; these data were analyzed by 1-way ANOVA and log-rank (Mantel-Cox) test.

### Locomotor activity

#### Apparatus

Locomotor activity (ambulation) was measured in Plexiglas test cages [480 x 480 x 400(h) mm] by a computer-operated, photocell-equipped apparatus (Motil, TSE, Bad Homburg, Germany). Photocells were 40-mm spaced. Test cages were located in a sound-proof, dimly-lit room adjacent to the housing room.

#### Experimental procedure

Experiment 4 evaluated the effect of acute and systemic treatment with different, relatively high doses of ISL on spontaneous locomotor activity. Rats were initially exposed to the homecage 2-bottle “alcohol (10% v/v) *vs* water” choice regimen with unlimited access for 24 hours/day throughout 10 consecutive days. Subsequently, rats were trained to lever-respond for alcohol using the same procedure described above. As a result, the “alcohol” history of these rats was identical to that of the rats used in Experiments 1A-D, 2, and 3.

This experiment employed a total of *n*=40 rats, divided into 4 groups of *n*=10 matched for body weight and number of responses on the alcohol lever over the last 3 self-administration sessions of the maintenance phase. The locomotor-activity test was conducted the day after completion of the maintenance phase and lasted 30 min. Rats were unfamiliar to the motility cage, in order to provide relatively high baseline levels of spontaneous locomotor activity (i.e., a desirable condition to amplify the possible suppressing effect of the tested drug) (see Kelley 1993). ISL was administered i.p. at doses of 0, 20, 40, and 80 mg/kg, 30 min before start of the locomotor-activity test. ISL dose range was remarkably higher than that used in Experiments 1 A, 2, and 3: this larger dose range was chosen to identify possible sedative and motor-incoordinating effects.

The measured variable in Experiment 4 was the number of motility counts (photocell breaks), recorded automatically by the apparatus. Data were divided into six 5-min time intervals and statistically analyzed by a 2-way (ISL dose; time interval) ANOVA with repeated measures on the factor “time”. The total (cumulated) number of motility counts over the entire 30-min session was statistically evaluated by 1-way ANOVA.

### Blood alcohol levels

#### Apparatus

Blood samples were analyzed by means of an enzymatic system [GL5 Analyzer (Analox Instruments, London, UK)] based on measurement of oxygen consumption in the alcohol-acetaldehyde reaction.

#### Experimental procedure

Experiment 5 evaluated the effect of acute and systemic treatment with different doses of ISL on BALs. Rats were initially exposed to the homecage 2-bottle “alcohol (10% v/v) *vs* water” choice regimen with unlimited access for 24 hours/day throughout 10 consecutive days. Subsequently, rats were trained to lever-respond for alcohol using the same procedure described above. Consequently, the “alcohol” history of these rats was identical to that of rats used in Experiments 1A-D, 2, and 3.

This experiment employed a total of *n*=40 rats, divided into 4 groups of *n*=10 matched for body weight and number of responses on the alcohol lever over the last 3 self-administration sessions of the maintenance phase. The experiment was conducted the day after completion of the maintenance phase. Food pellets were removed 4 hours before the experiment, to ensure that rats had empty stomachs at the time of alcohol infusion. ISL was administered i.p. at doses of 0, 5, 10, and 20 mg/kg; 30 min later, rats were treated i.g. with 1 g/kg alcohol (15% v/v). ISL dose range was chosen to be identical to that tested in Experiments 1 A, 2, and 3. Alcohol dose was chosen as known to produce intoxicating BALs in sP rats (e.g.: Colombo et al. 2014). Blood samples (50 μL) were collected from the tip of the tail of each rat at 30, 60, 120, and 240 min after alcohol administration.

The measured variable in Experiment 5 was BALs (expressed in mg%). Data on BAL time-course were statistically evaluated by 2-way (ISL dose; time) ANOVA with repeated measures on the factor “time”, followed by Tukey’s test for *post hoc* comparisons. Data on the area under the curve (AUC) of BAL time-course [expressed as (h*µg/ml)] were statistically evaluated by 1-way ANOVA, followed by Tukey’s test for *post hoc* comparisons.

## Results

### Testing systemic ISL on alcohol and sucrose self-administration under the FR schedule (Experiment 1 A)

**Fig. 2:**
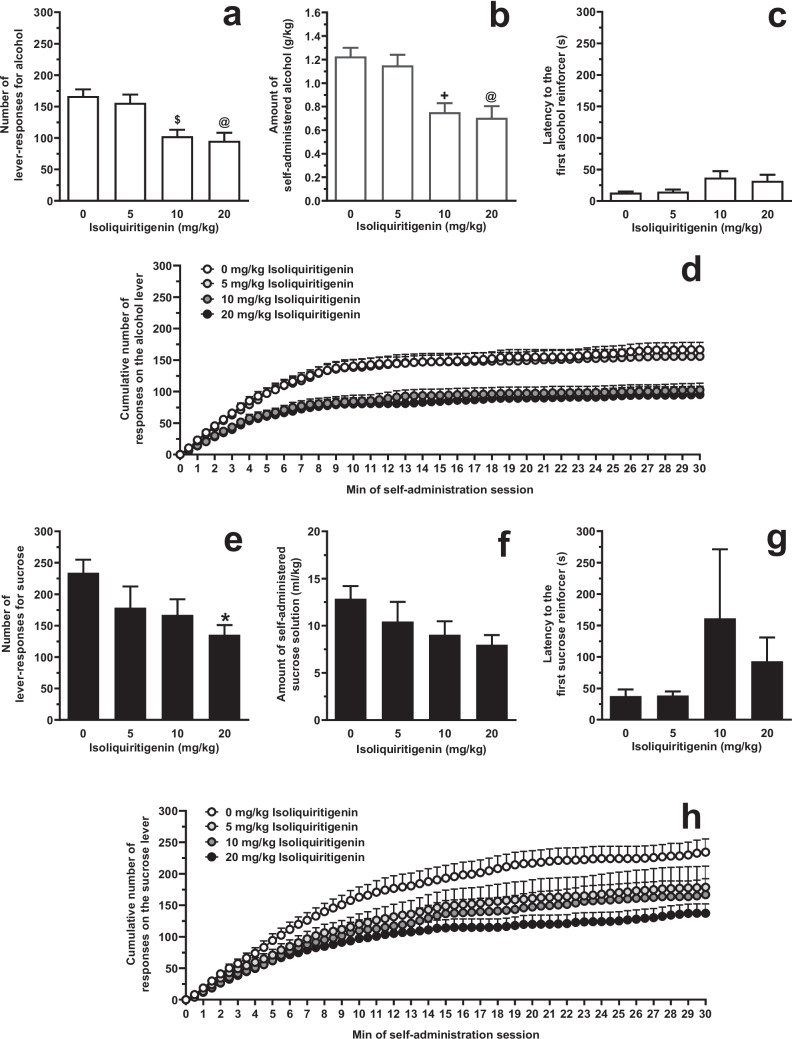
Effect of acute, systemic treatment with isoliquiritigenin (ISL) on (i) number of lever-responses for alcohol (panel **A**), amount of self-administered alcohol (panel **B**), latency to the first alcohol reinforcer (panel **C**), and cumulative response pattern of alcohol self-administration (panel **D**), and (ii) number of lever-responses for sucrose (panel **E**), amount of self-administered sucrose solution (panel **F**), latency to the first sucrose reinforcer (panel **G**), and cumulative response pattern of sucrose self-administration (panel **H**) in female Sardinian alcohol-preferring (sP) rats. Lever-responding for oral alcohol (15% v/v, in water) or sucrose (1% w/v, in water) occurred under the Fixed Ratio 5 schedule of reinforcement in daily 30-min self-administration sessions. In panels D and H, the self-administration session was divided into 30 intervals of 1 min each. Each bar or point is the mean ± SEM of *n*=14-16 rats.★: *P*<0.05, $: *P*<0.005, +: *P*<0.001, and @: *P*<0.0005 in comparison to the rat group treated with 0 mg/kg ISL (Tukey’s test)

Acute i.p. treatment with ISL reduced the number of lever-responses for alcohol [F(3,52)=9.30, *P*<0.0001; η^2^=0.35] in female sP rats exposed to the FR5 schedule of reinforcement (Fig. 2A). *Post hoc* analysis indicated that statistical significance was reached by treatment with 10 (*P*<0.005) and 20 (*P*<0.0005) mg/kg ISL. The magnitude of the reducing effect of 10 and 20 mg/kg ISL on number of lever-responses for alcohol averaged approximately 40% and 45%, respectively. Reduction in number of lever-responses for alcohol resulted in a proportional decrease in the amount of self-administered alcohol [F(3,52)=9.56, *P*<0.0001; η^2^=0.36] (Fig. 2B). At *post hoc* analysis, statistical significance was reached by treatment with 10 (*P*<0.001) and 20 (*P*<0.0005) mg/kg ISL. Treatment with ISL also increased latency to the first alcohol reinforcer [F(3,52)=9.82, *P*<0.05; η^2^=0.13], although no statistical significance between ISL doses was detected at *post hoc* analysis (Fig. 2C). Lever-responding for water was negligible (averaging <4 per session in all rat groups) and not altered by treatment with ISL (data not shown).

Acute i.p. treatment with ISL reduced the number of lever-responses for sucrose [F(3,60)=2.76, *P*<0.05; η^2^=0.12] in female sP rats exposed to the FR5 schedule of reinforcement (Fig. 2E). *Post hoc* analysis indicated that statistical significance was reached only by treatment with 20 mg/kg ISL (*P*<0.05). The magnitude of the reducing effect of 20 mg/kg ISL on number of lever-responses for sucrose averaged approximately 40%. Reduction in number of lever-responses for sucrose did not result in a statistically significant decrease in the amount of self-administered sucrose solution [F(3,60)=1.91, *P*>0.05; η^2^=0.09] (Fig. 2F). Treatment with ISL did not alter latency to the first sucrose reinforcer [F(3,60)=2.80, *P*>0.05; η^2^=−0.01] (Fig. 2G). Lever-responding for water was negligible (averaging <5 per session in all rat groups) and not altered by treatment with ISL (data not shown).

### Testing pretreatment with SCH50911 on the effect of systemic ISL on alcohol self-administration under the FR schedule (Experiment 1B)

**Fig. 3:**
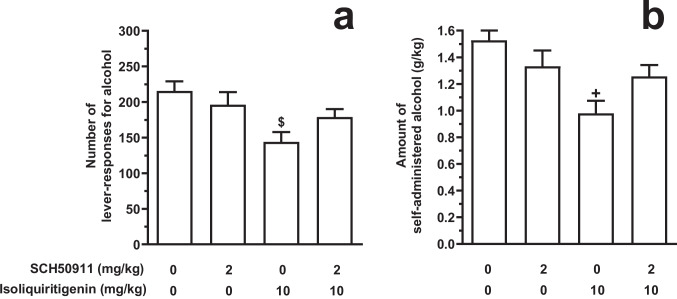
Effect of acute pretreatment with the GABA_B_ receptor antagonist, SCH50911, on the reducing effect of acutely administered isoliquiritigenin (ISL) on number of lever-responses for alcohol (panel **A**) and amount of self-administered alcohol (panel **B**) in Sardinian alcohol-preferring (sP) rats. Lever-responding for oral alcohol (15% v/v, in water) occurred under the Fixed Ratio 5 schedule of reinforcement in daily 30-min sessions. Each bar is the mean ± SEM of *n*=15 rats. $: *P*<0.005 and +: *P*<0.001 in comparison to the rat group treated with 0 mg/kg SCH50911 *plus* 0 mg/kg ISL (Tukey’s test)

Acute pretreatment with SCH50911 attenuated the reducing effect of acute, systemic administration of ISL on number of lever-responses for alcohol [F(3,56)=4.52, *P*<0.01; η^2^=0.19] in female sP rats exposed to the FR5 schedule of reinforcement (Fig. 3A). As expected, based on the results of Experiment 1 A, treatment with 10 mg/kg ISL reduced – in comparison to vehicle treatment – the number of lever-responses for alcohol by approximately 35% (*P*<0.005). Conversely, combination of 2 mg/kg SCH50911 (ineffective when given alone) with 10 mg/kg ISL resulted in an average value of number of lever-responses of alcohol that did not differ, at *post hoc* analysis, from that recorded in the vehicle-treated rat group. An identical pattern was observed in the amount of self-administered alcohol [F(3,56)=5.70, *P*<0.005; η^2^=0.23] (Fig. 3B). More specifically, treatment with 10 mg/kg ISL produced an approximately 35% reduction – in comparison to vehicle treatment – in amount of self-administered alcohol (*P*<0.001). Combination of 2 mg/kg SCH50911 (ineffective when given alone) with 10 mg/kg ISL resulted in an average value of amount of self-administered alcohol that did not differ, at *post hoc* analysis, from that recorded in the vehicle-treated rat group. Lever-responding for water was negligible (averaging <3 per session in all rat groups) and not altered by drug treatment (data not shown).

### Testing the combination of GS39783 and systemic ISL on alcohol self-administration under the FR schedule (Experiment 1 C)

**Fig. 4:**
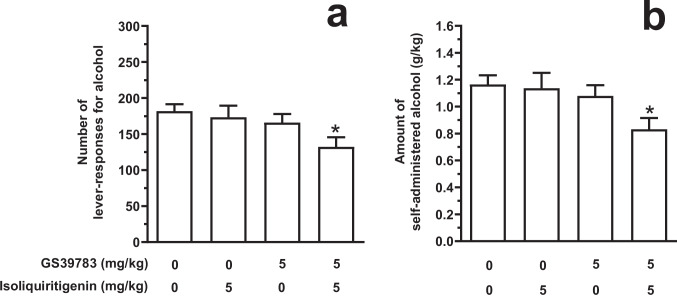
Effect of the acute combination of *per se* ineffective doses of the positive allosteric modulator of the GABA_B_ receptor, GS39783, and isoliquiritigenin (ISL) on number of lever-responses for alcohol (panel A) and amount of self-administered alcohol (panel B) in Sardinian alcohol-preferring (sP) rats. Lever-responding for oral alcohol (15% v/v, in water) occurred under the Fixed Ratio 5 schedule of reinforcement in daily 30-min sessions. Each bar is the mean ± SEM of *n*=14 rats. ★: *P*<0.05 in comparison to the rat group treated with 0 mg/kg GS39783 *plus* 0 mg/kg ISL (Tukey’s test)

Acute treatment with the combination of GS39783 and ISL reduced the number of lever-responses for alcohol [F(3,52)=2.83, *P*<0.05; η^2^=0.14] in female sP rats exposed to the FR5 schedule of reinforcement (Fig. 4A). Neither 5 mg/kg GS39783 nor 5 mg/kg ISL, when administered alone, altered the number of lever-responses for alcohol. Conversely, treatment with their combination resulted in an approximately 30% reduction – in comparison to vehicle treatment – in number of lever-responses for alcohol (*P*<0.05). Reduction in number of lever-responses for alcohol was associated to a proportional decrease in the amount of self-administered alcohol [F(3,52)=2.94, *P*<0.05; η^2^=0.15] (Fig. 4B). More specifically, neither 5 mg/kg GS39783 nor 5 mg/kg ISL, when given alone, altered the amount of self-administered alcohol; conversely, treatment with their combination resulted in an approximately 30% reduction – compared to vehicle treatment – in the amount of self-administered alcohol (*P*<0.05). Lever-responding for water was negligible (averaging <4 per session in all rat groups) and not altered by drug treatment (data not shown).

### Testing intragastric ISL on alcohol and sucrose self-administration under the FR schedule (Experiment 1D)

**Fig. 5:**
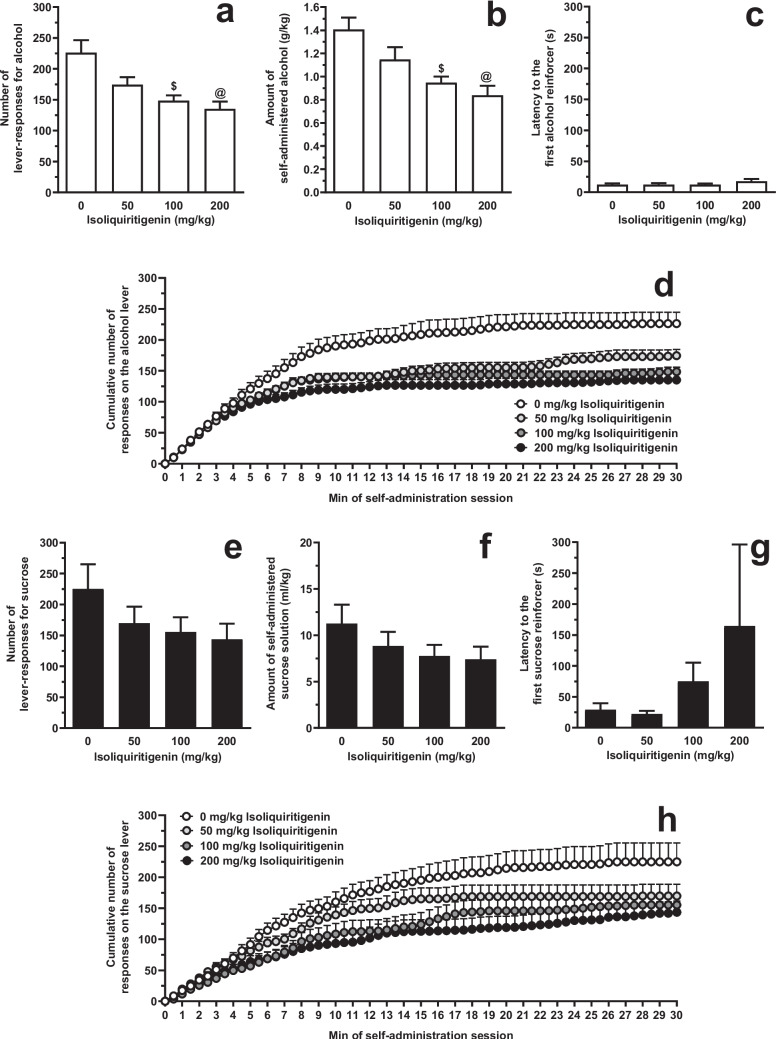
Effect of acute, intragastric treatment with isoliquiritigenin (ISL) on (i) number of lever-responses for alcohol (panel **A**), amount of self-administered alcohol (panel **B**), latency to the first alcohol reinforcer (panel **C**), and cumulative response pattern of alcohol self-administration (panel **D**), and (ii) number of lever-responses for sucrose (panel **E**), amount of self-administered sucrose solution (panel **F**), latency to the first sucrose reinforcer (panel **G**), and cumulative response pattern of sucrose self-administration (panel H) in female Sardinian alcohol-preferring (sP) rats. Lever-responding for oral alcohol (15% v/v, in water) or sucrose (1% w/v, in water) occurred under the Fixed Ratio 5 schedule of reinforcement in daily 30-min self-administration sessions. In panels D and H, the self-administration session was divided into 30 intervals of 1 min each. Each bar or point is the mean ± SEM of *n*=7-10 rats. $: *P*<0.005 and @: *P*<0.0005 in comparison to the rat group treated with 0 mg/kg ISL (Tukey’s test)

Acute i.g. treatment with ISL reduced the number of lever-responses for alcohol [F(3,36)=8.48, *P*<0.0005; η^2^=0.41] in female sP rats exposed to the FR5 schedule of reinforcement (Fig. 5A). *Post hoc* analysis indicated that statistical significance was reached by treatment with 100 (*P*<0.005) and 200 (*P*<0.0005) mg/kg ISL. The magnitude of the reducing effect of 100 and 200 mg/kg ISL on number of lever-responses for alcohol averaged approximately 35% and 40%, respectively. Reduction in number of lever-responses for alcohol resulted in a proportional decrease in the amount of self-administered alcohol [F(3,36)=7.95, *P*<0.0005; η^2^=0.40] (Fig. 5B). At *post hoc* test, statistical significance was reached by treatment with 100 (*P*<0.005) and 200 (*P*<0.0005) mg/kg ISL. Treatment with ISL did not alter latency to the first alcohol reinforcer [F(3,36)=0.96, *P*>0.05; η^2^=0.07] (Fig. 5C). Lever-responding for water was negligible (averaging <4 per session in all rat groups) and not altered by treatment with ISL (data not shown).

Acute i.g. treatment with ISL did not result in a statistically significant reduction of number of lever-responses for sucrose [F(3,24)=1.47, *P*>0.05; η^2^=0.16] (Fig. 5E), amount of self-administered sucrose solution [F(3,24)=1.22, *P*>0.05; η^2^=0.13] (Fig. 5F), and latency to the first sucrose reinforcer [F(3,24)=0.93, *P*>0.05; η^2^=0.10] (Fig. 5G) in female sP rats exposed to the FR5 schedule of reinforcement. Lever-responding for water was negligible (averaging <3 per session in all rat groups) and not altered by treatment with ISL (data not shown).

### Testing systemic ISL on alcohol and sucrose self-administration under the PR schedule (Experiment 2)

**Fig. 6:**
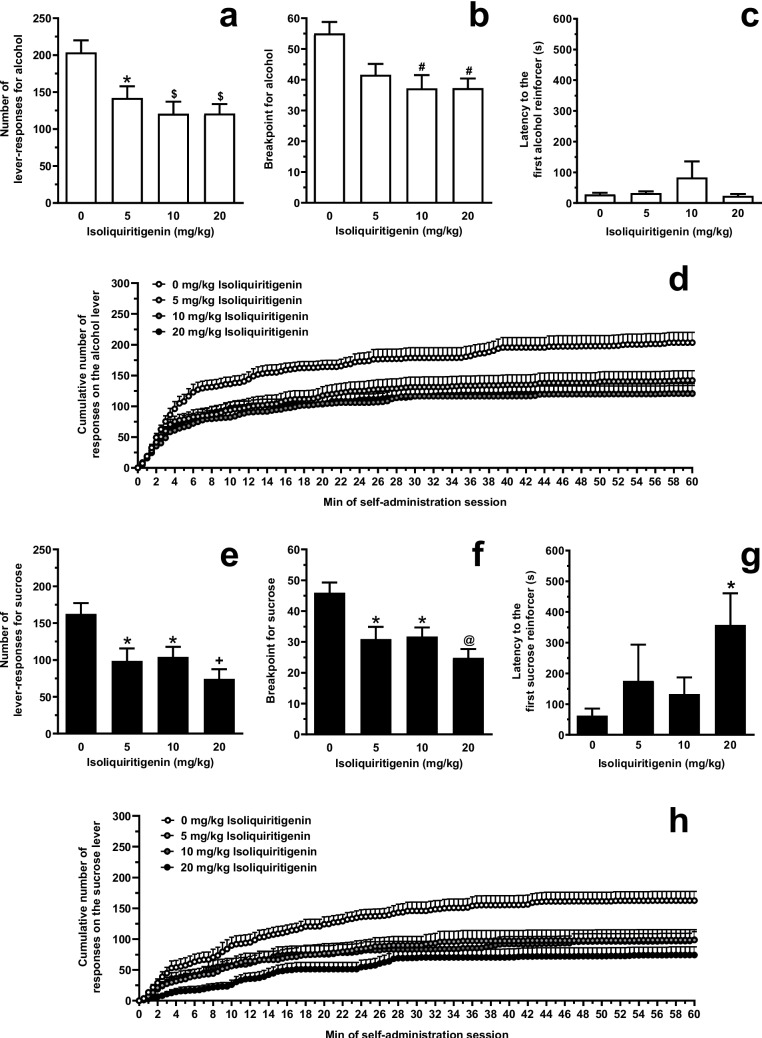
Effect of acute, systemic treatment with isoliquiritigenin (ISL) on (i) number of lever-responses for alcohol (panel **A**), breakpoint for alcohol (panel **B**), latency to the first alcohol reinforcer (panel **C**), and cumulative response pattern of alcohol self-administration (panel **D**), and (ii) number of lever-responses for sucrose (panel **E**), breakpoint for sucrose (panel **F**), latency to the first sucrose reinforcer (panel **G**), and cumulative response pattern of sucrose self-administration (panel **H**) in female Sardinian alcohol-preferring (sP) rats. Lever-responding for oral alcohol (15% v/v, in water) or sucrose (1% w/v, in water) occurred under a Progressive Ratio schedule of reinforcement, in which the response requirement (RR) was increased progressively over a 60-min session, with breakpoint being defined as the lowest RR not achieved by the rat. In panels D and H, the self-administration session was divided into 60 intervals of 1 min each. Each bar or point is the mean ± SEM of *n*=10-12 rats. ★: *P*<0.05, #: *P*<0.01, $: *P*<0.005, +: *P*<0.001, and @: *P*<0.0005 in comparison to the rat group treated with 0 mg/kg ISL (Tukey’s test in panels **A**, **B**, **E**, and **F**; Dunn’s test in panel **G**)

Acute i.p. treatment with ISL reduced the number of lever-responses for alcohol [F(3,44)=6.35, *P*<0.005; η^2^=0.30] in female sP rats exposed to the PR schedule of reinforcement (Fig. 6A). *Post hoc* analysis indicated that statistical significance was reached by treatment with all 3 doses of ISL (5 mg/kg: *P*<0.05; 10 mg/kg: *P*<0.005; 20 mg/kg: *P*<0.005). The magnitude of the reducing effect of 5, 10, and 20 mg/kg ISL on number of lever-responses for alcohol averaged approximately 30%, 40%, and 40%, respectively. Treatment with ISL also reduced breakpoint for alcohol [F(3,44)=5.10, *P*<0.005; η^2^=0.26] (Fig. 6B). At *post hoc* test, statistical significance was reached by treatment with 10 (*P*<0.01) and 20 (*P*<0.01) mg/kg ISL. The magnitude of the reducing effect of 10 and 20 mg/kg ISL on breakpoint for alcohol averaged approximately 30%. Conversely, treatment with ISL did not affect latency to the first alcohol reinforcer [F(3,44)=1.91, *P*>0.05; η^2^=−0.05] (Fig. 6C). Lever-responding on the inactive lever was negligible (averaging <5 per session in all rat groups) and not altered by treatment with ISL (data not shown).

Acute i.p. treatment with ISL reduced the number of lever-responses for sucrose [F(3,36)=6.45, *P*<0.005; η^2^=0.35] in female sP rats exposed to the PR schedule of reinforcement (Fig. 6E). *Post hoc* analysis indicated that statistical significance was reached by treatment with all 3 doses of ISL (5 mg/kg: *P*<0.05; 10 mg/kg: *P*<0.05; 20 mg/kg: *P*<0.001). The magnitude of the reducing effect of 5, 10, and 20 mg/kg ISL on number of lever-responses for sucrose averaged approximately 40%, 35%, and 55%, respectively. Treatment with ISL also reduced breakpoint for sucrose [F(3,36)=7.15, *P*<0.001; η^2^=0.37] (Fig. 6F). Statistical significance at *post hoc* analysis was reached by treatment with all 3 doses of ISL (5 mg/kg: *P*<0.05; 10 mg/kg: *P*<0.05; 20 mg/kg: *P*<0.0005). The magnitude of the reducing effect of 5, 10, and 20 mg/kg ISL on breakpoint for sucrose averaged approximately 40%, 35%, and 55%, respectively. Treatment with ISL increased latency to the first sucrose reinforcer [F(3,36)=7.45, *P*<0.05; η^2^=−0.08] (Fig. 6G). *Post hoc* test indicated that statistical significance was reached only by treatment with 20 mg/kg ISL (*P*<0.05). After treatment with 20 mg/kg ISL, latency to the first sucrose reinforcer was approximately 9 times higher than that recorded after vehicle treatment. Lever-responding on the inactive lever was negligible (averaging <5 per session in all rat groups) and not altered by treatment with ISL (data not shown).

### Testing systemic ISL on cue-induced reinstatement of alcohol seeking (Experiment 3)

**Fig. 7:**
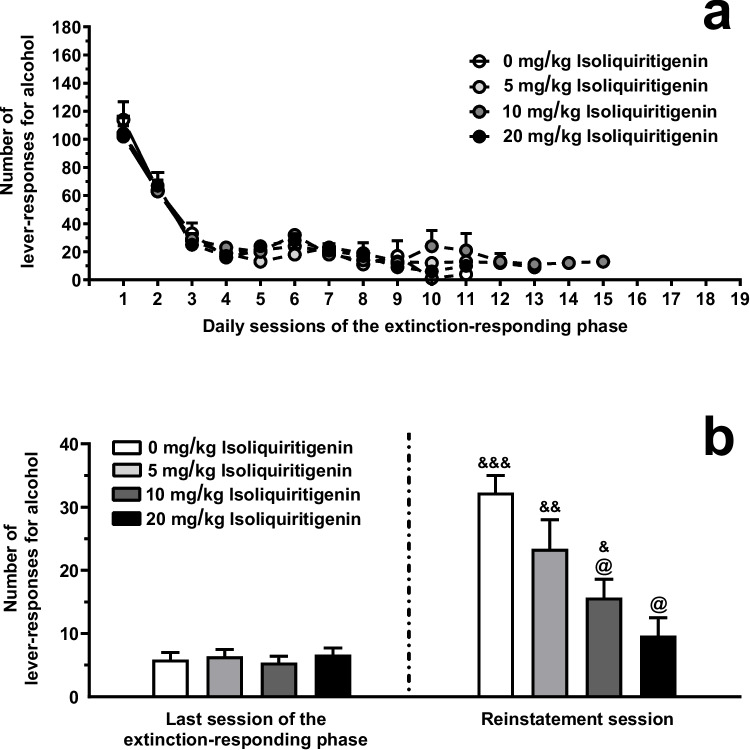
Effect of acute, systemic treatment with isoliquiritigenin (ISL) on cue-induced reinstatement of alcohol-seeking behavior in female Sardinian alcohol-preferring (sP) rats. After training to lever-responding for oral alcohol (15% v/v, in water) under the Fixed Ratio 5 schedule of reinforcement, rats were exposed to an extinction-responding phase (panel **A**) during which lever-responding was unreinforced. The reinstatement session occurred once rats achieved a given extinction criterion. In the reinstatement session (panel **B**, left portion), unreinforced lever-responding was resumed by presentation of alcohol-associated cues. The reinstatement session lasted 60 min. In panel A, each point is the mean ± SEM of an *n* value varying from 12 in the first extinction-responding sessions to 1-2 in some of the last extinction-responding sessions. In panel B, each bar is the mean ± SEM of *n*=12 rats. &: *P*<0.05, &&: *P*<0.0005, and &&&: *P*<0.0001 in comparison to the same rat group in the last session of the extinction-responding phase (Tukey’s test); @: *P*<0.0005 in comparison to the rat group treated with 0 mg/kg ISL in the reinstatement session (Tukey’s test)

Regarding the extinction-responding phase, Log-rank (Mantel-Cox) test indicated that the profile of lever-responding did not differ among the 4 groups of female sP rats subsequently treated with 0, 5, 10, and 20 mg/kg ISL and then exposed to the reinstatement session (χ^2^=1.70, *P*>0.05) (Fig. 7A). Additionally, the 4 rat groups did not differ in number of extinction-responding sessions needed to achieve the extinction criterion [7.7 ± 0.6, 7.4 ± 0.9, 9.2 ± 0.9, and 8.0 ± 0.7 (mean ± SEM) in rats subsequently treated with 0, 5, 10, and 20 mg/kg ISL, respectively; F(3,44)=1.05, *P*>0.05; η^2^=0.07].

Regarding the reinstatement session, ANOVA indicated significant effects of presentation of the alcohol-associated stimulus complex [F(1,44)=57.63, *P*<0.0001; η^2^=1.31] and treatment with ISL [F(3,44)=7.39, *P*<0.0005; η^2^=0.47], and a significant interaction [F(3,44)=7.11, *P*<0.0005; η^2^=0.48], on number of responses on the alcohol lever. Number of lever-responses during the last session of the extinction-responding phase was virtually identical in the 4 rat groups subsequently treated with 0, 5, 10, and 20 mg/kg ISL (Fig. 7B, left portion). In the reinstatement session (Fig. 7B, right portion), presentation of the alcohol-associated stimulus complex reinstated lever-responding in the vehicle-treated rat group: the number of lever-responses averaged approximately 35 and was approximately 5 times higher than that recorded in the same rat group during the last session of the extinction-responding phase (*P*<0.0001). Acute i.p. treatment with ISL suppressed lever-responding in the reinstatement session (Fig. 7B, right portion); *post hoc* test indicated that statistical significance was reached by treatment with 10 mg/kg (*P*<0.0005) and 20 mg/kg (*P*<0.0005) ISL. The magnitude of the suppressing effect of 10 and 20 mg/kg ISL on lever-responding averaged approximately 50% and 70%, respectively.

### Testing systemic ISL on spontaneous locomotor activity (Experiment 4)

**Fig. 8:**
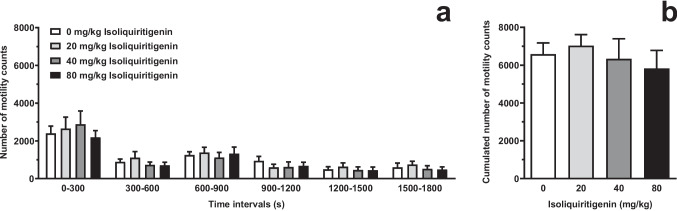
Effect of acute, systemic treatment with isoliquiritigenin (ISL) on spontaneous locomotor activity in female Sardinian alcohol-preferring (sP) rats. Rats were initially trained to lever-respond for oral alcohol (15% v/v, in water) under the Fixed Ratio 5 schedule of reinforcement and then exposed to a 30-min locomotor-activity session. The measured variable was the number of motility counts (photocell breaks) recorded by the apparatus. In panel A, data are expressed as mean ± SEM of number of motility counts in six 5-min time intervals in *n*=10 rats. In panel B, data are expressed as mean ± SEM of total (cumulated) number of motility counts over the entire locomotor-activity session in *n*=10 rats

Acute i.p. treatment with a series of relatively high doses of ISL did not alter, at any 5-min time-interval, the number of motility counts in female sP rats exposed to an open-field motility cage [F_dose_(3,36)=0.43, *P*>0.05, η^2^=0.10; F_time_(5,180)=29.07, *P*<0.0001, η^2^=0.81; F_interaction_(15,180)=0.34, *P*>0.05, η^2^=0.28] (Fig. 8A). Moreover, no difference was observed in the cumulated number of motility counts recorded over the entire 30-min locomotor-activity session [F(3,36)=0.37, *P*>0.05] (Fig. 8B).

### Testing systemic ISL on BALs (Experiment 5)

**Fig. 9:**
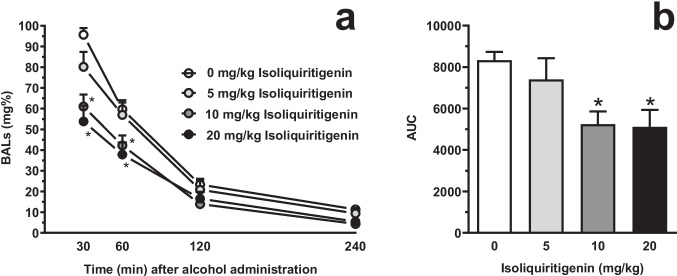
Effect of acute, systemic pretreatment with isoliquiritigenin (ISL) on blood alcohol levels (BALs) in female Sardinian alcohol-preferring (sP) rats. Rats were initially trained to lever-respond for oral alcohol (15% v/v, in water) under the Fixed Ratio 5 schedule of reinforcement and then treated intragastrically with 1 g/kg alcohol (15%, v/v). Blood samples were collected from the tip of the rat tail at different time point after alcohol administration. Each point or bar is the mean ± SEM of *n*=10 rats. In panel **A**, BALs were expressed in mg%; ★: *P*<0.05 in comparison to the rat group treated with 0 mg/kg ISL at the corresponding time (Tukey’s test). In panel **B**, data on the area under the curve (AUC) of BAL time-course are expressed as (h*µg/ml); ★: *P*<0.05 in comparison to the rat group treated with 0 mg/kg ISL (Tukey’s test)

Acute i.p. pretreatment with ISL reduced BALs produced in female sP rats by acute i.g. administration of 1 g/kg alcohol [F_dose_(3,36)=6.49, *P*<0.005, η^2^=0.81; F_time_(2.07,74.64)=302.60, *P*<0.0001, η^2^=8.40; F_interaction_(9,108)=4.88, *P*<0.0001, η^2^=0.41] (Fig. 9A). *Post hoc* analysis indicated that the reducing effect of ISL on BALs (i) was limited to the first 2 recording times (30- and 60-min), (ii) concerned the 2 highest doses of ISL (10 and 20 mg/kg), and (iii) was of a magnitude averaging – at both ISL doses – approximately 30% and 40% at the 30- and 60-min recording times, respectively.

Accordingly, pretreatment with ISL reduced AUC of BAL time-course [F(3,36)=4.47, *P*<0.01; η^2^=0.27] (Fig. 9B). *Post hoc* analysis indicated that statistical significance was reached by treatment with 10 (*P*<0.05) and 20 (*P*<0.05) mg/kg ISL. The magnitude of the reducing effect of 10 and 20 mg/kg ISL on AUC of BAL time-course averaged approximately 35% and 40%, respectively.

## Discussion

Data from Experiments 1 A, 2, and 3 indicate that acute, systemic treatment with ISL, a major active constituent of *Glycyrrhiza glabra* roots, reduced (i) operant oral alcohol self-administration under both FR and PR schedules of reinforcement (measures of the reinforcing and motivational properties of alcohol, respectively) and (ii) cue-induced reinstatement of alcohol-seeking behavior in selectively bred alcohol-preferring sP rats. In all three experiments, these effects occurred at doses of ISL as low as 10 mg/kg. In terms of efficacy, lever-responding for alcohol under both schedules of reinforcement was approximately halved by treatment with ISL, while magnitude of the reducing effect of ISL on reinstatement of alcohol seeking was remarkably greater, resembling – at the 20-mg/kg ISL dose – suppression of this relapse-like behavior.

Additional insights on ISL effect on alcohol self-administration come from the analysis of cumulative response patterns recorded in Experiments 1 A (FR schedule; Fig. 2D) and 2 (PR schedule; Fig. 6D): in comparison to vehicle treatment, treatment with ISL resulted in (i) less steep curves (suggestive of a reduced frequency in lever-responding for alcohol), and (ii) lower plateau values (suggesting that fewer ratios were completed before lever-responding for alcohol ended); conversely, and in agreement with data on latency to the first reinforcer, treatment with ISL exerted a very limited effect on lever-responding for alcohol over the first couple of min of the self-administration session, thus suggesting that ISL treatment did not impact on the rat urge to start lever-responding and consuming alcohol.

Treatment with ISL was also effective after intragastric infusion (Experiment 1D). The reducing effect of i.g. ISL on alcohol self-administration under the FR schedule of reinforcement occurred at the doses of 100 and 200 mg/kg, i.e. an order of magnitude higher than the potency observed after systemic injection. In spite of the different potency, cumulative response patterns of alcohol self-administration after i.g. treatment with ISL had features [i.e., less steep curves and lower plateau values when compared to vehicle treatment (Fig. 5D)] similar to those detected after systemic ISL treatment. While these data indicate that ISL ability to reduce alcohol self-administration is maintained after i.g. treatment, they are also suggestive of a reduced bioavailability when ISL is given *per os*. Future studies aimed at extending the present investigation to extracts of *Glycyrrhiza glabra* roots should also assess whether the plant roots contain ingredients with protective properties on ISL metabolism.

ISL effect on alcohol-related behaviors was specific, but not selective. Specificity was assessed evaluating the effect of ISL treatment on spontaneous locomotor activity (Experiment 4). To increase the reliability of data comparison, rats used in this experiment were previously exposed to an alcohol “history” similar to that of rats employed in the alcohol self-administration experiments. The collected results indicate that spontaneous locomotor activity was completely unaffected by systemic treatment with doses of ISL up to four times higher than those found to reduce lever-responding for alcohol and suppress reinstatement of alcohol seeking. These data suggest that the observed ISL-induced reduction of alcohol-related behaviors was likely not influenced by any possible motor-incoordinating or sedative effect of ISL.

Selectivity was assessed by investigating the effect of ISL treatment on self-administration of a sucrose solution. Experiments of alcohol and sucrose self-administration had an identical design, in terms of rat line and sex, experimental procedure, and even baseline levels of lever-responding (sucrose concentration was indeed calibrated as to result in numbers of lever-responses comparable to those sustained by alcohol). Systemic treatment with ISL decreased sucrose self-administration under both FR and PR schedules of reinforcement, with potency and efficacy not largely different from those recorded in the “alcohol” experiments (Experiments 1 A and 2). A modest degree of selectivity for alcohol self-administration was also seen after i.g. treatment with ISL (Experiment 1D). Features of ISL effect on sucrose self-administration are effectively recapitulated in the cumulative response patterns depicted in Figs. 2H, 5H, and 6H. These data, overall suggestive of the ability of ISL to lessen the reinforcing and motivational properties of sucrose, urge testing of the anorectic properties of ISL in more proper experimental models of excessive eating and food addiction.

As mentioned above, molecular-docking (Lin et al. 2021) and pharmacological (Jang et al. 2008; Lin et al. 2021) studies suggest that ISL binds to the GABA_B_ receptor and has agonistic properties. Experiments 1B and 1 C of the present study tested the hypothesis that the reducing effect of ISL on alcohol self-administration was indeed mediated by activation of the GABA_B_ receptor. Accordingly, pretreatment with a receptor antagonist (SCH50911; Experiment 1B) resulted in a partial blockade of ISL-induced reduction in alcohol self-administration. The partial, rather than complete, SCH50911-induced blockade of the reducing effect of ISL on alcohol self-administration could allude to (i) receptor systems other than the GABA_B_ receptor mediating ISL effect and/or (ii) higher doses of SCH50911, likely resulting in a more complete occupancy of the GABA_B_ receptor, are required to exert a larger prevention of ISL effect. The latter hypothesis is however difficult to test, as SCH50911 may *per se* reduce alcohol self-administration (Maccioni et al. 2022) and 2 mg/kg SCH50911 was among the highest ineffective doses (e.g.: Maccioni et al. 2016, 2017).

In the other “mechanistic” experiment, combination with a GABA_B_ PAM (GS39783; Experiment 1 C), given at a low and *per se* ineffective dose, clearly potentiated the effect of ISL (itself given at a *per se* ineffective dose) on alcohol self-administration. These results strengthen the notion that ISL behaves as a GABA_B_ receptor agonist, the *in vivo* effects of which are indeed potentiated by GABA_B_ PAMs (see Urwyler 2016).

If ISL behaves as a GABA_B_ receptor agonist, it is not surprising that ISL effects on alcohol-related behaviors have features that closely resemble those of baclofen. Baclofen has indeed been found to suppress operant alcohol self-administration under both FR (Petry 1997; Anstrom et al. 2003; Janak and Gill 2003; Besheer et al. 2004; Maccioni et al. 2005, 2012; Liang et al. 2006; Walker and Koob 2007; Dean et al. 2011; Lorrai et al. 2016; Williams et al. 2016; Haile et al. 2021; Domi et al. 2023; Jeanblanc et al. 2023) and PR (Walker and Koob 2007; Maccioni et al. 2008b, 2012) schedules of reinforcement as well as reinstatement of alcohol seeking (Maccioni et al. 2008a; Vengeliene et al. 2018) in rats and mice. Similarities extend to (i) GABA_B_ PAM-induced potentiation, as the GABA_B_ PAMs, CGP7930, GS39783, and *rac*-BHFF, potentiated the reducing effect of baclofen on alcohol self-administration in rats (Liang et al. 2006; Maccioni et al. 2015), and (ii) limited selectivity, as baclofen treatment reduced sucrose self-administration in rats at the same doses that reduced alcohol self-administration (Anstrom et al. 2003; Janak and Gill 2003; Maccioni et al. 2005, 2008b; Echeverry-Alzate et al. 2021).

Data from Experiment 5 indicate that systemic pretreatment with ISL reduced BALs generated by the acute i.g. administration of 1 g/kg alcohol. This effect was evident over the first hour after alcohol administration (corresponding to 90 min after ISL injection), while it vanished at subsequent recording times. GABA_B_ receptors located in the gastrointestinal tract (Nakajima et al. 1996; Castelli et al. 1999; see Hyland and Cryan 2010) might be the neural substrate underlying this ISL effect: their activation would indeed interfere with gastric emptying and/or intestinal motility (e.g.: Ong and Kerr 1982; Kaneko et al. 2010; Auteri et al. 2014; see Hyland and Cryan 2010), possibly altering alcohol absorption and metabolism. However, it should be noted that baclofen had never been found to decrease BALs in rats and mice (Broadbent and Harless 1999; Barreto Zaleski et al. 2001; Boehm et al. 2002; Arias et al. 2009), thus suggesting that ISL effect on BALs might occur *via* mechanisms of action other than agonism at the GABA_B_ receptor.

Research on ISL and alcohol seems to be still relatively unexplored. The results of the present study add indeed to few and sparse literature data, limited – as to our knowledge – to (i) protection against alcohol-induced hepatic steatosis in AML-12 cells (Na et al. 2018) and (ii) attenuation, after intracerebellar ISL injection, of alcohol-induced ataxia in mice (Al-Rejaie and Dar 2006).

The results of the present study agree with a line of research suggesting that ISL may exert anti-addictive properties. More specifically, acute i.g. treatment with ISL prevented (i) cocaine-induced hyperlocomotion (Jeon et al. 2008) [an effect reproduced by the ISL isoform, liquiritigenin (Jang et al. 2011)], (ii) anxiety-related behaviors associated to nicotine withdrawal (Wang et al. 2020), and (iii) nicotine-induced locomotor sensitization (Wang et al. 2020) in rats. Acute i.g. treatment with ISL also abolished cocaine-stimulated increase in extracellular dopamine in the rat nucleus accumbens (Jang et al. 2008), i.e. a major neurochemical correlate of the rewarding properties of several drugs of abuse (see Koob and Volkow 2010). As mentioned above, this specific ISL effect was blocked by pretreatment with SCH50911 (Jang et al. 2008).

Consistent data were collected in studies testing extracts of *Glycyrrhiza glabra* roots, in which active ingredients other than ISL may of course account for the observed pharmacological effects. More specifically, methanolic extracts of *Glycyrrhiza glabra* roots abolished locomotor sensitization produced by repeated treatment with methamphetamine (Zhao et al. 2014a) and nicotine (Zhao et al. 2017) as well as development and expression of methamphetamine-induced conditioned place preference (Zhao et al. 2014a) in rats; notably, the extract effect on methamphetamine-induced locomotor sensitization was completely prevented by treatment with SCH50911 (Zhao et al. 2014a). Finally, treatment with a methanolic extract of roots from *Glycyrrhiza uralensis*, another medicinal plant belonging to *Glycyrrhiza* genus and comprising ISL among its active ingredients, attenuated methamphetamine-induced hyperlocomotion in rats (Zhao et al. 2014b).

We recognize that this study has several limitations. A first, major limitation is represented by testing only female sP rats. Future studies testing ISL effect on alcohol self-administration and reinstatement of alcohol seeking in male sP rats will fill this gap and allow us to assess whether sex differences exist in ISL potency and efficacy in attenuating alcohol-related behaviors in sP rats. At present, and based on the results of previous experiments suggesting limited differences in the reducing effect of GABA_B_ receptor ligands between female and male sP rats (Lorrai et al. 2019), we might predict that sensitivity of alcohol-related behaviors to the reducing effects of ISL will be similar between female and male sP rats. Secondly, focusing solely on acutely administered ISL represents another major limitation of the present study. Studies are now needed to investigate whether and to what extent the reducing effects of ISL on alcohol-related behaviors are maintained after repeated treatment or, alternatively, if and to what extent tolerance may develop to the reducing effects of repeatedly administered ISL on alcohol-related behaviors. Thirdly, the results of Experiment 1D currently represent no more than a proof-of-concept of the efficacy of intragastrically administered ISL on alcohol-related behaviors. Additional studies are now required to qualify in greater detail this ISL attribute, including investigations on larger dose ranges and specificity. Finally, future studies should investigate whether receptor systems other than the GABA_B_ receptor contribute to mediating ISL effects on alcohol-related behaviors and alcohol metabolism. The composite pharmacological profile of ISL (see Peng et al. 2015; Ramalingam et al. 2018; Mustafa et al. 2025) may offer hints for possible additional mechanisms requiring proper scrutiny.

In conclusion, the results of the present study confirm that ISL is a naturally occurring, *in vivo* effective GABA_B_ receptor agonist; they also extend the pharmacological profile of ISL to suppression of different alcohol-related behaviors in rats, closely replicating a major feature of baclofen pharmacology. Broadly speaking, these results are in line with the notion that medicinal plants may represent a plentiful source of new and potentially effective GABA_B_ receptor ligands (see Colombo 2024).

## Data Availability

Raw data will be available upon request.
